# Comparison of surgical outcomes between early and advanced class of jugular paragangliomas following application of our modified surgical techniques

**DOI:** 10.1038/s41598-023-27821-y

**Published:** 2023-01-17

**Authors:** Peng Zhao, Yibo Zhang, Feng Lin, Dedi Kong, Yisi Feng, Chunfu Dai

**Affiliations:** 1 Department of Otology and Skull Base Surgery, Eye Ear Nose and Throat Hospital, Fudan University, Shanghai, 200031 China; 2 Key Laboratory of Hearing Medicine, Ministry of Health, Eye Ear Nose and Throat Hospital, Fudan University, Shanghai, 200031 China; 3 Department of Otolaryngology Head and Neck Surgery, Shenzhen Baoan Women’s and Children’s Hospital, Jinan University, Shenzhen, 518102 China; 4 Department of Otolaryngology Head and Neck Surgery, Affiliated Nanping First Hospital, Fujian Medical University, Nanping, 353000 China

**Keywords:** Surgical oncology, Head and neck cancer

## Abstract

To compare the safety and effectiveness of surgical treatment of jugular paragangliomas (JPs) following the application of our modified surgical techniques. Fifty-six patients with JPs were analyzed for tumor classification, surgical outcomes, and intratumor blood vessels. The gross total resection in C1–2 (100%) was significantly greater than that in C3 and D (66.7%). Good postoperative facial nerve (FN) function (House–Brackmann I–II) was achieved in 89.5% C1–2 cases, which was not significantly different from C3 and D (93.3%) (*P* = 0.694). Preoperative and postoperative lower cranial nerve (LCN) deficits correlated with the Fisch’s classification of tumors (*P* < 0.05), and intraoperative blood loss was greater in advanced tumors (*P* = 0.050). Further study showed that the cross-sectional area of intratumor blood vessels was positively associated with intraoperative blood loss (*P* < 0.001). Surgical excision of JPs is a safe and effective strategy, and early surgical treatment is a good option for patients with C1–2 tumors without surgical contraindications.

## Introduction

Head and neck paragangliomas (HNPs) are rare slow-growing, benign, highly vascularized tumors, but are locally destructive lesions, accounting for only 0.6% of all head and neck tumors^1^. Jugular paragangliomas (JPs) are the most common primary neoplasms in the jugular foramen, arising from paraganglia cells located within the adventitia of the dome of the jugular bulb. Although histologically considered benign, JPs represent locally aggressive, destructive neoplastic lesions and close proximity to the facial nerve (FN), lower cranial nerves (LCNs), internal carotid artery (ICA), jugular bulb, posterior fossa, cochlea and labyrinthine, and surgical management of JPs is challenging^2,3^. In rare cases, JPs can secrete catecholamines and metastasize to lymph nodes and distant organs, which significantly worsens the disease prognosis^1,4^.

There has been intensive debate over the various treatment options for JPs, including surgical excision, radiotherapy and wait-and-scan^3,5,6^. Currently, due to a thorough understanding of the anatomic and surgical approaches of the skull base and various modified surgical approaches, technological improvements in microsurgery, neuromonitoring, and neuroradiology have made surgical removal of paragangliomas in the jugular foramen easier and safer^7–12^. According to the Fisch classification of temporal bone paragangliomas (TBPs)^13,14^, TBPs are traditionally classified into classes A, B, C, and D based on the location and extension assessed by high-resolution computed tomography (HRCT) and magnetic resonance imaging (MRI) of the temporal bone. The Fisch class A and B tumors can be treated surgically by skillful otologists with standard approaches with almost no complications, preserving the FN and inner ear function and offering the patient a complete cure^15^. Fisch class C or D JPs are still a great challenge to skull base surgeons, and cranial nerve injury is the main source of postoperative morbidity. In recent years, our group has developed two improved surgical techniques, tunnel packing or push packing of the inferior petrous sinus and tension-free FN anterior rerouting, that have resulted in significant control of bleeding from the inferior petrous sinus and improved postoperative FN function^11,12^. The purpose of this study was to compare the safety and effectiveness of surgical treatment of JPs between early-stage and advanced-stage tumors following the application of our modified surgical techniques and to propose a reasonable strategy to manage JPs.

## Materials and methods

This was a retrospective review of 56 patients with JPs diagnosed and treated at the Eye Ear Nose and Throat Hospital of Fudan University (Shanghai, China) between October 2010 and January 2021. Patients who were managed with radiotherapy or wait-and-scan protocols were not enrolled in this study. This study was approved by the Ethics Committee of the Eye Ear Nose and Throat Hospital of Fudan University (No. 2021048). Our study involving human research participants had been performed in accordance with the Declaration of Helsinki. At our center, all patients underwent temporal-bone HRCT and gadolinium-enhanced MRI, and 24 of 56 patients underwent magnetic resonance arteriography (MRA) and magnetic resonance venography. Digital subtraction angiography was performed to identify the feeding arteries, as well as to assess ICA involvement and contralateral venous drainage, and then preoperative endovascular embolization was carried out on all patients to minimize intraoperative bleeding. Blood catecholamine levels were routinely evaluated preoperatively. The diagnostic work-ups for the postoperative evaluation of clinical signs associated with LCN deficits include laryngoscopy, shoulder and tongue movement. The Fisch classification was used for staging tumors^13,14^. Preoperative and postoperative FN function were graded according to the House-Brackmann grading system^16^.

The infratemporal fossa approach type A (IFTA-A) was employed in all the patients, and the surgical procedures have been described elsewhere^14,17,18^. The IFTA-A with either anterior transposition or nerve grafting of the FN was applied in the present study. If the epineurium was intact, we performed FN transposition. If the tumor invades the FN and the perineurium was infiltrated by the tumor, the FN was sacrificed and a simultaneous FN grafting is performed with the great auricular nerve. Several modifications were made to reduce FN tension during FN anterior transposition, which we defined as tension-free FN anterior rerouting^12^. (1) The FN, digastric muscle and parotid gland were anteriorly displaced without dissecting the FN within the parotid gland. The upper parotid gland was then sutured tightly to the lower margin of the temporal muscle to shorten the distance from the geniculate ganglion to the FN in the stylomastoid foramen. (2) A long articulated retractor was placed at a 45° angle to push the posterior belly of the digastric muscle and parotid gland anteriorly and superiorly to further shorten the distance of the facial nerve from the genicular ganglion to the stylomastoid foramen. To control bleeding from the inferior petrous sinus and to better preserve LCN function, the sigmoid sinus tunnel packing or push packing technique with surgical and intrabulbar dissection was applied intraoperatively ^11,19^.

**Figure 1:**
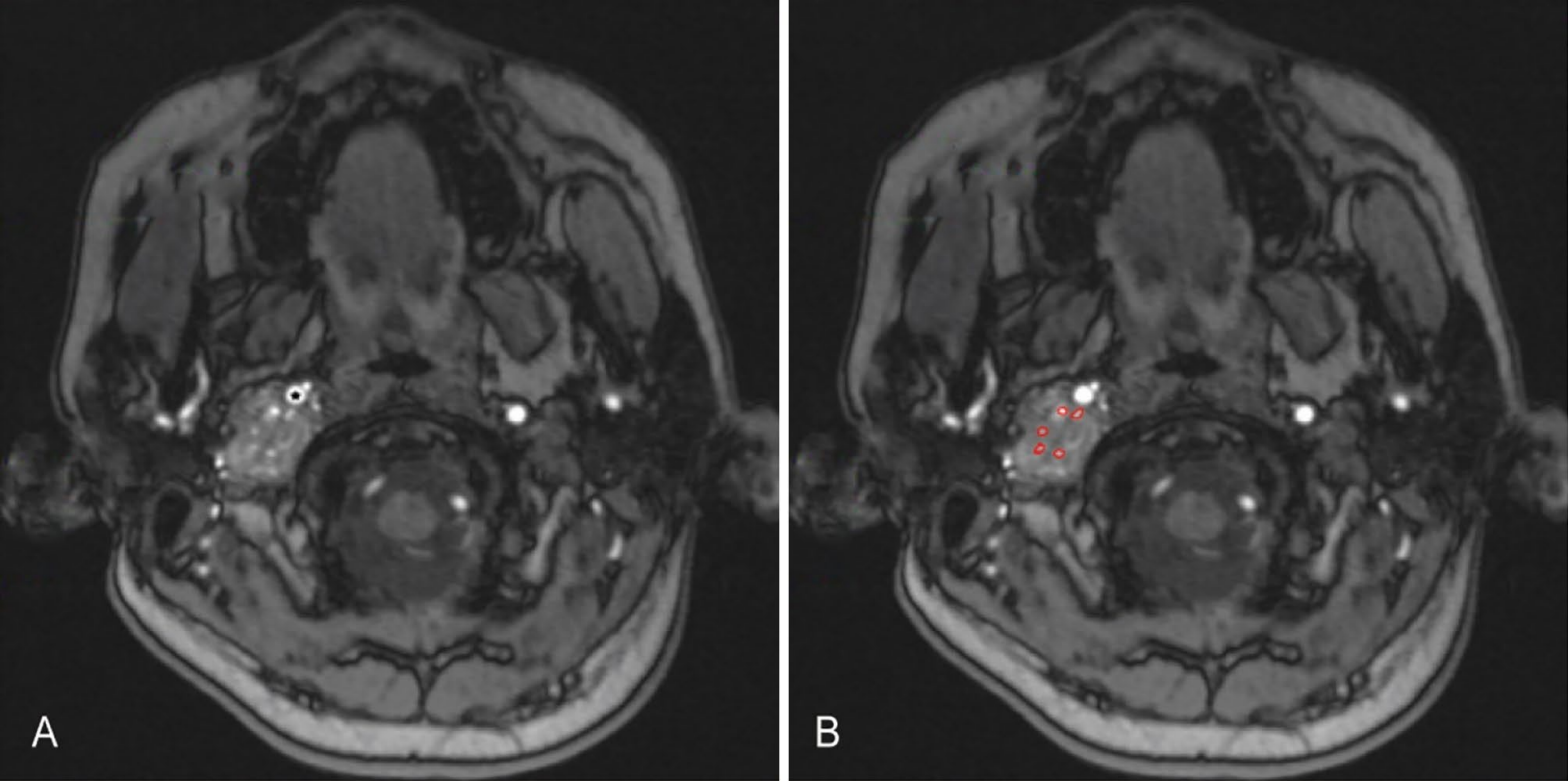
The preoperative axial MRA image of patient with C3 JPs on the right. There are multiple intratumor blood vessels and intraoperative blood loss was 600 ml. (**A**) The black star indicates the internal carotid artery. (**B**) The red areas represent the maximum cross-sectional area of intratumor blood vessels.

**Figure 2:**
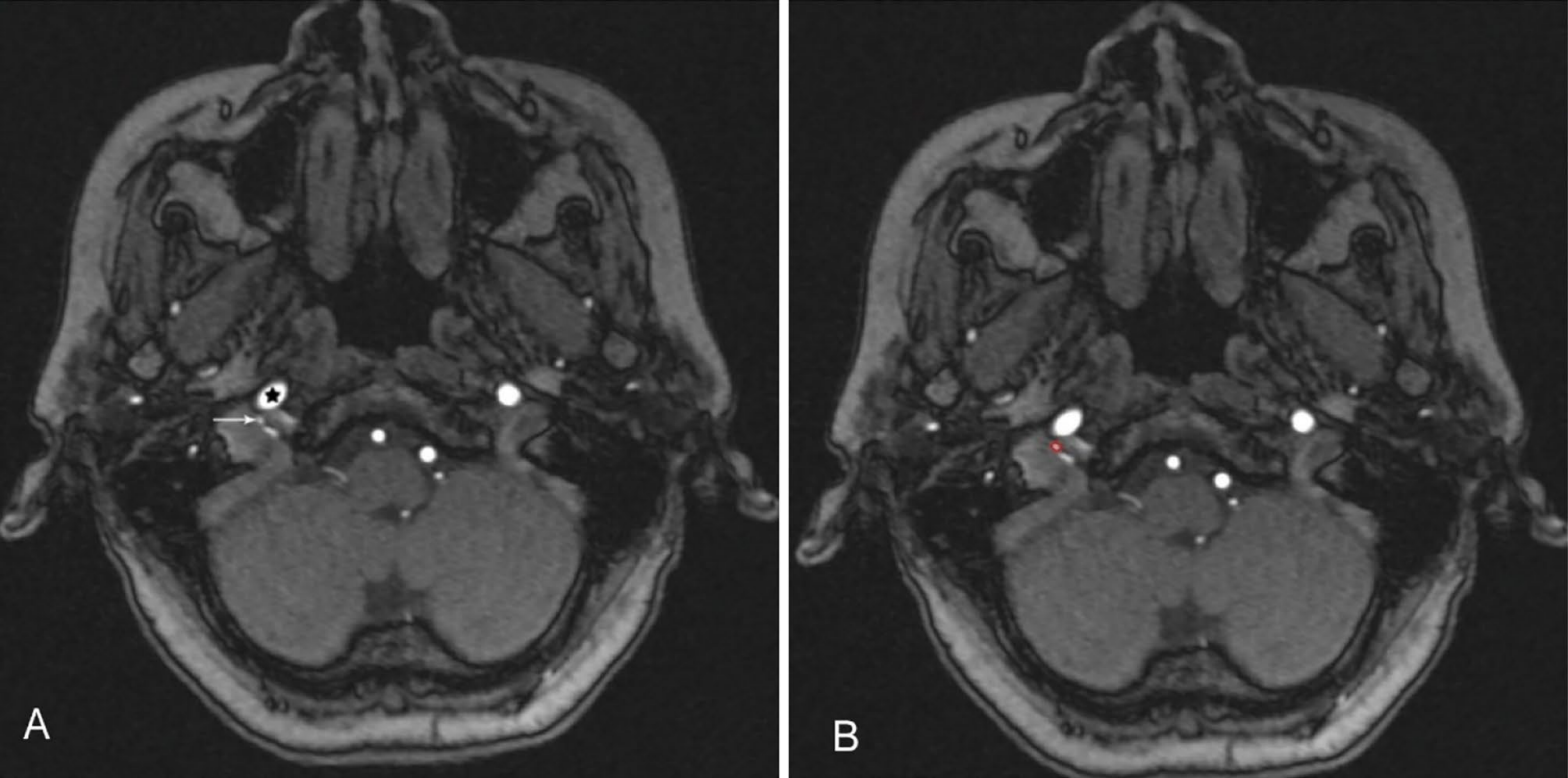
The preoperative axial MRA image of patient with C2 JPs on the right. There is only one intratumor blood vessel and intraoperative blood loss was 100 ml. The black star indicates the internal carotid artery. (**A**) The white arrow indicates intratumor blood vessel. (**B**) The red area represents the maximum cross-sectional area of intratumor blood vessel.

To identify whether the intratumor blood vessels were correlated with intraoperative blood loss and the Fisch classification of the tumor, preoperative MRA images were evaluated by a surgeon and a senior radiologist. In the axial plane, we selected the maximum cross-sectional area with intratumor blood vessels (Figs. 1, 2) and applied ImageJ software to measure the maximum cross-sectional area of intratumor blood vessels. We measured and summed the cross-sectional area of all intratumor blood vessels if there were multibranch arteries in the tumor.

Intraoperative management of the FN, LCNs and inferior petrosal sinus was noted, and blood loss and surgical complications were reviewed. Gross total resection (GTR) and recurrence were assessed by gadolinium-enhanced MRI. Follow-up duration was defined as the period from surgery to the most recent office visit or patient contact. Follow-up included physical examination and temporal-bone MRI with enhancement. MRI with enhancement was usually performed at a minimum of 3 months postoperatively and annually thereafter in our hospital.

In addition, to analyze the efficiency of surgical treatment of classes C1, C2, C3 and D, we pooled C1-4 De1-2/Di1-2 tumors as class D tumors. We defined C1 and C2 tumors as the early class and C3 and D tumors as the advanced class. The Chi-square test was applied to compare surgical outcomes among different groups using SPSS version 21.0 (SPSS Inc., Chicago, IL, USA). Spearman’s correlation was used to analyze the correlation between the Fisch classification of the tumor, intraoperative blood loss and the maximum cross-sectional area of the feeding artery. *P*-values < 0.05 were considered to be statistically significant.

### Ethics declarations

Our study involving human research participants had been performed in accordance with the Declaration of Helsinki. Informed consent was obtained from all subjects and/or their legal guardian(s).

## Results

### Demography

A total of 56 patients with JPs underwent surgery at our center between October 2010 and January 2021. Of the 56 patients, the mean age of the cohort was 45.5 years, and 18 (32.1%) patients were male and 38 (67.9%) were female. Eight patients presented with bilateral or multiple HNPs, including two with ipsilateral carotid body tumor (CBT), four with ipsilateral vagal paragangliomas (VP), and one with contralateral CBT. In one patient, JPs coexisted with bilateral CBT, while in another patient, JPs coexisted with contralateral CBT and adrenal pheochromocytoma. No patient was diagnosed with malignant JP in this cohort. The mean follow-up duration was 29.5 months (range, 6–94 months).

### Clinical signs and symptoms

The clinical features of patients with JPs are summarized in Table 1. The most common symptoms were hearing loss (43 patients, 76.8%) and pulsatile tinnitus (42 patients, 75%). Fifteen (26.8%) patients presented with FN palsy and 20 patients (35.7%) presented with LCN palsy. Fifty patients (89.3%) had a red tumor behind the eardrum or in the external auditory canal. All patients had one or more symptoms at the initial diagnosis.

**Table 1:** Preoperative characteristics of patients with JPs.

Parameter	No of patients (n = 56, %)
Demographics	
Female	38 (67.9)
Male	18 (32.1)
Left	29 (51.8)
Right	27 (48.2)
Bilateral	8 (14.3)
Age, years (mean)	23–76 (45.5)
Symptoms	
Pulsatile tinnitus	42 (75)
Aural fullness	11 (19.6)
Ear pain	4 (7.1)
Otorrhea	2 (3.6)
Conductive hearing loss	4 (7.1)
Sensorineural hearing loss	39 (69.6)
Facial palsy	15 (26.8)
Signs	
IX CN deficit	1 (1.8)
X CN deficit	10 (17.8)
XI CN deficit	1 (1.8)
XII CN deficit	8 (14.3)
Reddish mass behind eardrum	26 (46.4)
External auditory canal mass	24 (42.9)

*CN* cranial nerve.

### Classification of tumors

In the present study, 4 (7.1%) patients were classified as C1 according to the Fisch classification, 25 (44.6%) as C2, 14 (25%) as C3, and 13 (23.2%) as D. Of them, 7 (12.5%) cases were of extradural extension and 6 (10.7%) were of intradural extension.

### FN management and FN function

All 56 tumors were removed via IFTA-A. The preoperative, postoperative FN function and intraoperative management of FN were shown in Table 2. There was no significant difference of FN between early-stage and advanced-stage tumors either preoperatively or postoperatively. Thirty-four patients with HB-I FN function preoperatively were confirmed with intact epineurium intraoperatively. Nineteen out of 34 patients were of C1-2, 15 patients were of C3-D. Of them, 17 patients (89.5%) had preserved good FN function (HB I–II) in classes C1 and C2 tumors postoperatively, while 14 patients (93.3%) had good FN function (HB I–II) in classes C3 and D tumors. There was no significant difference in FN function between the two groups (*χ*^2^ = 0.155, *P* = 0.694) (Table 2).

**Table 2:** Preoperative, postoperative FN function and intraoperative management of FN (n = 56).

Group	No	Preoperative FN function (n, %)		Management of FN							Postoperative FN function (n, %)	
		HB I–II	HB III–VI	TAR		TF-TAR	TF-PAR	FB	Resection of FN with graft	Resection of FN without graft	HB I–II	HB III–VI
C1–C2	29	23	6 (20.7)	5		10	4	1	7	2	17	12 (41.4)
C3–D	27	18	9 (33.3)	1		14	0	0	8	4	14	13 (48.2)
Total	56	41	15 (26.8)	6		24	4	1	15	6	31	25 (44.6)

*FN* facial nerve, *TAR* total anterior rerouting, *TF-TAR* total anterior rerouting with tension-free FN rerouting technique, *TF-PAR* partial anterior rerouting with tension-free FN rerouting technique, *FB* fallopian bridge of facial nerve.

### Preoperative and postoperative LCN dysfunction

Fourteen patients (25%) suffered from at least one LCN deficit in the preoperative evaluation. The presence of preoperative LCN deficits was correlated with the Fisch classification of tumors (Table 3), which showed a lower incidence of LCN dysfunction in classes C1 and C2 (3 cases, 10.3%) and poorer outcomes of LCN dysfunction in classes C3 and D (11 cases, 40.7%) (*χ*^2^ = 5.364, *P* = 0.021). The presence of postoperative LCN deficits in classes C3 and D (15 cases, 55.6%) was obviously higher than that in classes C1 and C2 (7 cases, 24.1%) (*χ*^2^ = 5.364, *P* = 0.033). Furthermore, CN X was the most commonly affected LCN, followed by CN XII.

**Table 3:** Preoperative and postoperative LCNs deficit (n = 56).

Group	No	Preoperative deficits of LCNs (n, %)	Preoperative individual LCNs deficit				Postoperative deficits of LCNs (n, %)	Postoperative individual LCNs deficits			
			IX	X	XI	XII		IX	X	XI	XII
C1–C2	29	3 (10.3)	0	3	0	2	7 (24.1)	3	4	5	4
C3–D	27	11 (40.7)	0	8	1	6	15 (55.6)	2	11	2	9
Total	56	14 (25)	0	11	1	8	22 (39.3)	5	15	7	13

*LCNs* lower cranial nerves.

### Correlation among Fisch classification of tumors, intraoperative blood loss and the maximum cross-sectional area of intratumor blood vessels

The estimated blood loss averaged 502 ml (100 to 2500 ml) in 56 patients. The mean intraoperative blood loss was 358.6 ml in class C1–2 tumors and 657.4 ml in class C3 and D tumors respectively. More intraoperative blood loss was not statistically correlated with advanced Fisch classification (*r* = 0.404, *P* = 0.050). Further study showed that the cross-sectional area of intratumor blood vessels was positively associated with intraoperative blood loss (*r* = 0.791, *P* < 0.001); the greater the cross-sectional area of intratumor blood vessels was, the more the intraoperative blood loss would be. However, a more advanced Fisch classification was not correlated with the maximum cross-sectional area of intratumor blood vessels (*r* = 0.285, *P* = 0.177) (Table 4).

**Table 4:** The correlation among Fisch classification of tumor, intraoperative blood loss and the maximum cross-sectional area of intratumor blood vessels.

Group	No	Intraoperative blood loss (ml)	The maximum cross-sectional area of intratumor blood vessels (cm^2^)
C1–C2	6	258.3 ± 91.7	0.112 ± 0.107
C3–D	18	511.1 ± 540.0	0.151 ± 0.093

### Tumor removal, residuals and recurrences

Gross total tumor removal was achieved in 47 cases (83.9%), and subtotal removal was achieved in 9 cases (16.1%). The GTR rate in classes C1 and C2 (100%) was significantly greater than that in classes C3 and D (66.7%). Three patients complained of recurrence, including two patients with Class D tumors, and one with C3 tumors. One patient underwent surgery again. The other 2 patients treated with radiotherapy reported no tumor progression during follow-up.

### Complications

One patient suffered from postoperative cerebrospinal fluid (CSF) leakage and underwent an additional procedure to repair the CSF leakage. Two patients had wound infections, which were successfully treated after surgical debridement. Two patients developed mild cerebral infarction postoperatively and recovered after conservative medical treatment without sequelae. No patients underwent tracheotomy or nasal feeding postoperatively. There were no perioperative or postoperative vascular injuries or deaths in the present series.

## Discussion

Although considered histologically benign, surgical management of JPs is challenging due to their infiltrative nature and proximity to important neurovascular structures. The Fisch class A and B tumors can be treated surgically by skillful otologists with standard approaches with rare complications, preserving the FN and inner ear function and offering the patient complete cure^15,20^. The management of the Fisch class C and D tumors remains controversial, and there are still various opinions, such as surgery, radiotherapy or wait-and-scan^3,6,21–26^. This study retrospectively analyzed the clinical efficacy of surgical treatment of Fisch class C and D tumors in our institution during the past 10 years following the application of our modified surgical techniques and concluded that surgical treatment of JPs is a safe and effective strategy, especially for C1 and C2 tumors.

In the case of surgical management of JPs, IFTA-A, with the permanent anterior rerouting of the FN and exposure of the infratemporal course of the ICA as described by Fisch in 1977, is considered as the standard procedure. Due to permanent anterior FN transposition, the FN might lose most of its extrinsic vascularity, resulting in a certain degree of facial paralysis after surgery. Advances in neuroimaging, skull base techniques and modified surgical approaches have markedly reduced the incidence of FN dysfunction during the last two decades^7–9,11,12,27^; however, a majority of surgeons are still concerned about postoperative FN function. In this study, preoperatively, six patients (20.7%) of classes C1 and C2 had HB grade III–VI, while nine (33.3%) had HB grade III–VI in classes C3 or D. Although there was no significant difference in FN function between the two groups, the incidence of facial paralysis in classes C3 and D were greater than that in classes C1 and C2 preoperatively. Thirty-four patients with normal FN function preoperatively and the epineurium were identified as intact intraoperatively in our study. They underwent tension-free FN anterior rerouting, and 31 cases (91.2%) achieved good facial nerve function (House-Brackmann grade I–II), which indicated that good FN function was achieved in more than 90% of patients who underwent tension-free FN anterior rerouting. Wang et al. reported a postoperative rate of new FN deficits of 51.7% in eighty-nine patients with Fisch class C or D JPs^28^. In a recent study, 185 patients with Fisch class C or D JPs were treated surgically by Prasad and colleagues, and forty-three (23.2%) new FN deficits (House-Brackmann grade III–VI) were observed as a consequence of FN mobilization in IFTA-A^3^. In the present study, 31 patients (91.2%) achieved good facial nerve function following the application of tension-free FN anterior rerouting. The tension-free FN anterior rerouting technique significantly reduced the incidence of new FN deficits.

In our clinical practice, achieving optimal exposure of the jugular foramen while minimizing the damage to neurovascular structures and obtaining proximal and distal exposures of the ICA are the key points. The management of FN plays an important role in exposing of the jugular foramen. FN management strategies are included in the fallopian bridge technique and total or partial tension-free FN anterior rerouting technique, which are determined according to the extent of the lesion shown on temporal bone CT and MRI preoperatively. In the present study, most of the patients underwent tension-free FN anterior rerouting in cases of normal facial nerve function preoperatively, thereby reducing the incidence of FN dysfunction, and 31 patients (91.2%) achieved good facial nerve function postoperatively.

Given highly vascularized tumors, heavy intraoperative bleeding frequently occurs during the removal of JPs. To reduce intraoperative bleeding, preoperative super-selected embolization of the main feeding arteries of the tumor is routinely performed on patients with JPs. In addition, effectively controlling intraoperative bleeding from the inferior petrous sinus can provide a clearer surgical field and decrease the incidence of neurovascular structure damage. In the present study, we applied our modified technique, the sigmoid sinus tunnel packing and push packing technique, to control bleeding from the inferior petrous sinus^11^, which led to a mean blood loss of 502 ml.

Function preservation of LCNs is another key point that draws attention to the discussion of JP management options. The intrabulbar dissection technique was proposed in 2002 and was routinely applied to dissect tumors in our clinical practice as long as the tumor itself had not penetrated the medial wall of the jugular bulb or infiltrated the LCNs^19^. Prasad et al. noticed that the presence of intradural extension is usually associated with infiltration of the LCNs^3^. Grinblat et al. reported that early and limited C1 and C2 tumors without medial wall invasion reduces the risk of dysfunctionality of LCNs^29^. Thus resection of early JPs (classes C1-C2), where the lateral aspect of the jugular bulb is involved and the medial wall is not infiltrated, makes preservation of the LCNs possible. Our investigation also revealed a lower incidence (3 cases, 10.3%) of LCN dysfunction in classes C1 and C2 and a greater incidence (11 cases, 40.7%) in classes C3 and D preoperatively. Furthermore, surgery resulted in newly developed LCN deficits in 14.3% of patients. In addition, conservative surgical management of JPs with targeted subtotal resection based on the extent and location of tumors yields lower new-onset LCNs^21,23,30^. That is, the more tumor that is removed, the less likely the residual tumor is to grow^31^. Bacciu et al. demonstrated that class C1 and C2 tumors can be removed completely using IFTA-A with fewer LCN deficits^32^. Our results were consistent with a previous study, and GTR (100%) was able to achieve fewer postoperative new LCN deficits (4 cases, 13.8%) in classes C1 and C2. Therefore, radical excision is recommended for early-stage JPs to reduce the incidence rate of LCN deficits and prevent tumor recurrence instead of subtotal resection.

It has also been demonstrated that MR arteriography is not only useful to visualize the major feeding arteries of paragangliomas but is also helpful in the detection and characterization of paragangliomas^33^. Our study retrospectively reviewed the patient's preoperative MRA data and analyzed the correlation between the Fisch classification of tumors, intraoperative blood loss and the maximum cross-sectional area of intratumor blood vessels. We concluded that the advanced the more tumor class, the more intraoperative blood loss and the greater the cross-sectional area of intratumor blood vessels. However, there was no significant correlation between the Fisch classification of tumors and the maximum cross-sectional area of intratumor blood vessels. It is well known that the Fisch classification is based on the relationship of the tumor location with the ICA and intracranial extension, rather than the size of the tumor. The Fisch classification of tumors was positively correlated with intraoperative blood loss, indicating more bleeding in advanced tumors, resulting in unclear surgical fields, less GTR and increased injury of neurovascular structures. Therefore, we recommend early surgical intervention to reduce the incidence of perioperative complications and improve the GTR rate.

In recent years, radiotherapy has become popular as the first-line treatment for JPs due to its high tumor control and low morbidity compared with the surgical management of JPs^5,22,34^. Recently, Patel and colleagues reported a series of 40 cases with JPs treated by linear accelerator stereotactic radiosurgery^22^. Similar to the results of previous radiotherapy studies, the authors did not classify tumors according to the Fisch classification but preferred to report them based on tumor size. Considering that the mean pretreatment volume size was 8.9 cm^3^ (range 3.8–13.1 cm^3^), most of them were probably small tumors (possibly even class B tympanomastoid paragangliomas according to the Fisch classification), which can be treated surgically by skillful otologists with standard approaches with almost no complications and achieve gross total tumor removal. With improvements in surgical techniques and perioperative management, the incidence of cranial nerve injury and serious complications caused by surgical treatment has gradually decreased. However, the unique complications of radiotherapy cannot be avoided, such as temporal bone necrosis, brain necrosis, secondary malignancy and pituitary/hypothalamic insufficiency^34^. The most significant effect of radiotherapy is related to radiation‑induced fibrosis with obliteration of the vascular supply and not to direct destruction of tumor cells. Thus, it is important to re‑emphasize that radiotherapy only achieves tumor control and not a cure. Jansen et al. noted that the age of presentation was a risk factor for tumor growth, and 59 patients with JPs who underwent wait-and-scan with a median follow-up of 63.6 months showed that the age of presentation was an independent predictor of tumor growth and had a significant inverse correlation with growth rates: the younger the age of presentation, the greater the growth rate^6^. It is essential to achieve cure rather than tumor control in those who have more than 30 years of life expectancy. If radiotherapy is considered in young patients, it also means life-long surveillance scans and the psychological burden caused by living with the knowledge of having a residual tumor. Long-term treatment (surgery, radiotherapy or a combined modality) outcomes of 93 Fisch class C and D tumors were evaluated by Jansen’s group in 2018. One independent predictor of treatment outcome was reported: if treatment was delayed until tumor growth occurred, the chance of functional recovery was lower^21^. Taken together, we consider it important to make an early diagnosis and perform surgery as early as possible. As outlined above, although the management of JPs is challenging, we believe that surgical gross total resection of the tumor remains the mainstay of treatment of JPs.

## Conclusion

The low incidence of tumor residuals, tumor recurrences, facial paralysis, LCN deficits and other surgical complications in this study demonstrated the safety and efficacy of surgical excision of Fisch class C and D tumors, especially tumors in the early stage following the application of our modified surgical techniques. Consequently, we advocate that early surgical treatment is a good option for Fisch class C1 or C2 patients with no contraindications.

## Data Availability

The datasets generated and/or analyzed during the current study are available from the corresponding author on reasonable request.
